# Integrative Analysis of Metabolomic and Transcriptomic Data Reveals the Antioxidant Potential of Dietary Lutein in Chickens

**DOI:** 10.3389/fvets.2022.906853

**Published:** 2022-06-23

**Authors:** Tuanhui Ren, Wujian Lin, Shizi He, Xiuxian Yang, Mingjian Xian, Zihao Zhang, Wen Luo, Qinghua Nie, Xiquan Zhang

**Affiliations:** ^1^Department of Animal Genetics, Breeding and Reproduction, College of Animal Science, South China Agricultural University, Guangzhou, China; ^2^Guangdong Provincial Key Lab of Agro-Animal Genomics and Molecular Breeding, and Key Laboratory of Chicken Genetics, Breeding and Reproduction, Ministry of Agriculture, Guangzhou, China

**Keywords:** chicken, lutein, antioxidant, transcriptome, metabolome

## Abstract

Lutein can increase the body's skin color and has antioxidant potential. However, how it affects lipid metabolism and oxidative stress in chickens remains unknown. In this study, 74-day-old male chickens raised on feed supplemented with lutein had higher hip, back, breast, leg, shin and abdominal fat yellowness than the control group, and the livers of chickens in the lutein group had higher superoxide dismutase and glutathione peroxidase and lower malondialdehyde activities. To clarify the potential regulatory network regulated by lutein, we used RNA-seq and nontargeted metabolomics to detect changes in the male chicken liver and plasma, respectively. A total of 243 differentially expressed genes were significantly enriched in cytokine–cytokine receptor interaction signaling pathways, among others. A total of 237 significantly different metabolites were enriched in lysine biosynthesis and degradation and glycerophospholipid metabolism signaling pathways, among others. Finally, we comprehensively analyzed metabolome and transcriptome data and found that many differentially expressed genes and significantly different metabolites play crucial roles in lipid metabolism and oxidative stress. In summary, dietary lutein can improve male chicken skin yellowness and antioxidant indices and affect liver gene expression and plasma metabolites and may help improve the health of chickens.

## Introduction

Animals cannot synthesize carotenoids de novo; therefore, carotenoids in animals must be procured from food or partially modified through metabolic reactions (1). Lutein, an oxygen-containing carotenoid, is a xanthophyll lacking vitamin A activity. Its content is high in the macular area of the eye (2). Carotenoids increase the body's skin color, have antioxidant potential and anti-inflammatory activity, improve the immune response and prevent chronic diseases (3–5). The oral administration of lutein and zeaxanthin can improve human skin tone and induce whitening, which may be due to the antioxidant activity of carotenoids (6). Previous studies have shown that after feeding mice 100 and 250 mg (per kilogram body weight) of lutein for 1 month, superoxide dismutase (SOD), catalase (CAT), glutathione-*S*-transferase, glutathione peroxidase (GSH-PX) and glutathione (GSH) activities increased significantly (7). Oxidative stress induced by H_2_O_2_ reduces the viability of acute retinal pigment epithelial cells and increases total cell apoptosis and reactive oxygen species (ROS) production. However, lutein can protect cells from oxidative stress-induced damage (8). Some carotenoids can not only function as antioxidants by eliminating ROS but also participate in redox homeostasis in cells by regulating the nuclear factor-erythroid factor 2-related factor 2 and nuclear factor kappa B systems (9). The administration of 40 mg of lutein can significantly reduce the quantity of lipid peroxidation product (MDA) in rats with myocardial infarction induced by isoproterenol and significantly upregulate the protein expression of nuclear factor-erythroid factor 2-related factor 2 and heme oxygenase-1 (10).

A negative correlation exists between dietary lutein and serum lutein levels and obesity (11, 12). Lutein supplementation can improve liver lipid accumulation and insulin resistance induced by a high-fat diet in rats; lutein accelerates lipolysis and inhibits fat production by activating sirtuin 1 expression and regulating sirtuin 1-mediated pathways, limiting lipid accumulation in 3T3L cells and reducing rat abdominal fat (13, 14). In poultry studies, lutein is primarily used for the pigmentation of chicken skin and egg yolk. In countries such as China and Mexico, consumers' preference for yellow chickens is the primary business driver, and most consumers believe that this appearance is related to the health and freshness of the poultry (15–17). Incidentally, birds and humans have the same enzymes to metabolize dietary xanthophylls; thus, birds serve as a good nonprimate model to study xanthophyll metabolism (18). When lutein was fed to Arbor Acres broilers for 42 days, the color intensity of the shank and b^*^ value (yellowness) of the pectoralis and leg muscles significantly increased compared with those in the control group; the weight of abdominal fat decreased, but the difference was not significant (19). Adding lutein to the diet can increase the antioxidant capacity of the serum and liver in breeding hens and reduce lipid peroxidation in the serum and intestinal mucosa (20). Carotenoids act as antioxidants to neutralize ROS that cause oxidative stress and accelerate aging and disease (21). Because of this activity, carotenoids can prevent oxidative stress from damaging lipids, proteins and DNA (22). Recent studies have found that any factor that affects the balance of oxidation in the human body may change the content of carotenoids (23).

The liver is the hub of metabolism that plays a major role in lipid synthesis, degradation, and transport. However, whether lutein can affect the molecular regulation of liver lipid metabolism in chickens remains unclear. Lutein feeding can rapidly increase the concentration of carotenoids in animal plasma or serum, but no study has investigated the metabolite changes after feeding chickens lutein (24). In the present study, we aimed to explore the effects of lutein supplementation on skin yellowness, antioxidant properties, plasma metabolites and liver gene expression in chickens. First, the skin phenotype and liver antioxidant indices of yellow-feather chickens fed lutein were evaluated. Second, the nontargeted metabolomics approach was used to identify the significantly different metabolites (SDMs) in chicken plasma after lutein was added to the feed, and the differentially expressed genes (DEGs) in chicken liver were identified by RNA-seq to understand the molecules related to the antioxidant function of lutein. Finally, using online tools to comprehensively analyze the metabolome and transcriptome data, many SDMs and DEGs were found to play critical roles in lipid metabolism and oxidative stress.

## Materials and Methods

### Experimental Animals and Feeding

In total, 270 healthy male yellow-feather chickens at the age of 1 day from the same batch and with the same genetic background were transferred to the animal experiment base of South China Agricultural University. All the chickens were raised in cages under the same environment. The birds were fed commercial diets from Day 1 to Day 53 and then were randomly divided into three groups (no difference was found in the average weight among the groups). Three different dietary supplement groups were basal diet (BD), BD + 1 g/kg lutein (from marigold, 2%) and BD + 1.5 g/kg lutein (from marigold, 2%; Leader Bio-Technology CO., Ltd., Guangzhou, China). Each group (90 birds/group) was further subdivided into six cages (15 birds/cage). The birds were 53 days old at the beginning of the experiment and 74 days old at the end. The experimental period lasted 21 days. Recent studies have shown that supplementation with 20 and 40 mg/kg of lutein in the diet of chicks (the experimental period was 26 days) improved the jejunal morphology and cecal microbiota composition of yellow-feathered broilers under LPS stress (25). Mixed feeding of natural lutein (10%, 200 mg/kg) and canthaxanthin (50 mg/kg) to broilers (the experimental period was 35 days) significantly improved the skin yellowness value of broilers (26). These studies indirectly proved that the age of the chickens, lutein dose and experimental period were reasonable in this experiment.

The basal diet was formulated according to the Nutrient Requirements of Broilers (United States, NRC 1994). The composition of the basal diets for chickens is shown in Supplementary Table S1.

### Sample Collection and Measurement of Skin Color

Basal diet samples (powder and pellets) were collected for 53 days before and after pelleting to determine the content of natural lutein; the experimental group diet supplemented with lutein collected on Day 53 was tested for its natural lutein content before and after pelleting. The animals were weighed on days 53, 60, 67 and 74 after fasting for 12 h. Ten chickens were selected randomly from each group, and plasma samples were collected and slaughtered. The color of the hip, back, leg, shin, breast and abdominal fat were measured using a colorimeter (3NH, Guangzhou, China). Liver and skin tissue samples were collected from six birds in each group on Day 74 and were stored at −80°C until further analysis.

### Determination of ALT and AST

Blood was collected in a tube containing EDTA as an anticoagulant, the contents were centrifuged at 1,200 g for 10 min at 4°C, and plasma was stored at −80°C. The alanine aminotransferase (ALT) and aspartate aminotransferase (AST) activities in the plasma were measured using the corresponding test kits (Rayto, Shenzhen, China) according to the manufacturer's protocols.

### Determination of the Oxidation Index

The activities of SOD, GSH-PX and MDA in the liver tissues from each experimental group were measured according to the instructions of the SOD, GSH-PX and MDA assay kits (Jiancheng, Nanjing, China), respectively.

### Hematoxylin-Eosin Staining

Chicken liver tissues were obtained from the control group and lutein 2 group at 74 days. The tissues were quickly collected and fixed in 10% formalin and washed with PBS after 24 h. The fixed tissue was embedded in paraffin, sectioned, and stained with hematoxylin and eosin. Finally, the tissue sections were observed.

### Determination of the Lutein Content

Samples of basal diets and lutein diets (powder and granules) were tested for the content of natural lutein by HPLC (Guangzhou Huibiao Testing Technology Center).

### RNA Sequencing and Data Analysis

The livers of chickens were harvested for RNA sequencing (four samples per group). The processed samples were qualified and sequenced on an Illumina NovaSeq™ 6000 (LC-Bio Technology CO., Ltd., Hangzhou, China) with a read length of 300 bp (±50 bp) paired-end reads.

The reads contaminated with adaptor sequences were removed using Cutadapt software (version: cutadapt-1.9). We downloaded the Galgal 6 chicken reference genome and gene annotation files in the Ensembl database (http://asia.ensembl.org/Gallus_gallus/Info/Index). Next, low-quality bases and undetermined bases were removed, and HISAT2 software (version: hisat2-2.0.4) was used to map clean reads to the chicken reference genome. The mapped reads of each sample were assembled using StringTie software (version: stringtie-1.3.4d. Linux_x86_64) with default parameters. Next, Gffcompare software was used to merge the transcriptomes of all samples to reconstruct a comprehensive transcriptome (version: gffcompare-0.9.8.Linux_x86_64). After the comprehensive transcriptome was generated, StringTie and Ballgown (*R* package) were used to estimate the expression levels of all transcripts and determine the expression level for mRNAs by calculating FPKM [FPKM = (total_exon_fragments/mapped_reads (millions) × exon_length (kB))]. The DEGs were selected with fold change >2 or fold change <0.5 and *P-value* < 0.05 using DESeq2 package (http://www.bioconductor.org/packages/release/bioc/html/DESeq2.html) (27).

Gene Ontology (GO) has three ontology including molecular function, cellular component and biological process. Term is the basic unit of GO, and each term corresponds to an attribute. The GO database (http://www.geneontology.org/) was used to screen out the GO terms with significantly enriched DEGs (*P* < 0.05). In order to further explore the biological functions of the DEGs and the enriched metabolic pathways, the Kyoto Encyclopedia of Genes and Genomes (KEGG) database (https://www.kegg.jp/) was used to screen out the pathways with significantly enriched DEGs (*P* < 0.05). Top 20 GO terms and 20 pathways with the smallest *P*-value in the enrichment analysis results were selected to construct a scatter plot (*P* < 0.05).

### Quantitative Real-Time PCR (qRT–PCR)

Total RNA was isolated from the livers of chickens using a HiPure Unviersal RNA Mini Kit (Magen, Guangzhou, China), and reverse transcription was performed using a cDNA reverse transcription kit (Vazyme, Nanjing, China) according to the manufacturer's protocol. Relative expression was calculated using the 2^−Δ*ΔCt*^ method, and significance was determined using Student's *t*-test. All reactions were performed using four biological and three technical repetitions. Primers for the genes and internal control β-actin are presented in Supplementary Table S2.

### Metabolite Extraction

Blood was collected in a tube containing EDTA as an anticoagulant, followed by centrifugation at 1,200 g for 10 min at 4°C, and storage of plasma at −80°C. For analysis, the collected plasma samples were thawed on ice, and 20 μl of each was extracted with 120 μl of prechilled 50% methanol, vortexed for 1 min, and allowed to stand at room temperature for 10 min. After overnight incubation at −20°C, the extraction mixture was centrifuged at 4,000 g for 20 min, and the supernatant was transferred to a 96-well plate. Additionally, 10 μl was removed from each sample and mixed into a quality control (QC) sample. Before LC–MS analysis, all the samples were stored at −80°C.

### LC–MS Analysis

Chromatographic separation was performed using a UPLC system (SCIEX, UK). The analytes were separated on a Waters Acquity UPLC HSS T3 column (2.1 × 100 mm, 1.8 μm) maintained at 35°C. A TripleTOF5600plus high-resolution tandem mass spectrometer (SCIEX, UK) was used to detect metabolites eluted from the chromatographic column, and Q-TOF was run in positive and negative ion modes. During the entire collection period, the mass accuracy was calibrated every 20 samples. In addition, one QC sample was analyzed for every eight samples to evaluate the stability of the LC-MS. The raw LC–MS data and all metabolite molecules detected in the sample were analyzed by LC-Bio (Hangzhou, China).

### Metabolomics Data Processing

XCMS software was used to preprocess the LC–MS data and convert the original data files into the mzXML format, and then XCMS, CAMERA and metaX in R software were used to process the metabolites. Each ion was identified by its retention time and *m*/*z*. The intensity of each peak was recorded, and a three-dimensional matrix containing any specified peak index (retention time-*m*/*z* pairs), sample name (observed value), and ion intensity information (variable) was generated. Next, the information was matched using internal and public databases. The open access KEGG database and Human Metabolome Database (HMDB) were used to match accurate molecular mass data (*m*/*z*) with data in the databases and annotate metabolites within a threshold of 10 ppm. MetaX was used to further preprocess the peak intensity data. Features detected in more than 50% of QC samples or more than 80% of test samples were deleted, and the *k*-nearest neighbor algorithm was used to extrapolate the value of missing peaks to further improve data quality. Principal component analysis (PCA) was performed to detect outliers and batch effects using preprocessed datasets. The PQN algorithm was used to normalize the sample data, and then the QC sample was used to perform a QC-based robust LOESS (locally estimated scatterplot smoothing) signal correction. Additionally, the relative standard deviations of metabolic characteristics were calculated for all QC samples, and ions with standard deviations >30% were removed.

Using univariate analysis of fold-change and Student's *t*-test, BH correction was performed to obtain the *q*-value, combined with multivariate statistical analysis to obtain VIP (variable important for the projection) values using partial least squares-discriminant analysis (PLS-DA) and screen SDMs (ratio ≥2 or ratio ≤ 1/2; a *q*-value ≤ 0.05; VIP ≥1). Finally, pathway enrichment analysis of SDMs was performed using the KEGG database (http://www.kegg.jp/).

### Joint Analysis

The online OmicStudio tools (https://www.omicstudio.cn) were used to analyze clustering correlation heatmaps and connected networks of SDMs and DEGs. The correlation between skin yellowness and the antioxidant index and the correlation between skin yellowness and SDMs were analyzed using online OmicStudio tools.

### Statistical Analysis

Statistical analysis of the data was performed using Statistical Package for the Social Sciences (SPSS) 22.0 (IBM, Armonk, New York). One-way analysis of variance with *post hoc* test was used for multiple group comparisons, Student's *t*-test was used for two-group comparisons (^*^P < 0.05, ^**^P < 0.01), and the results were expressed as means ± standard deviation.

## Results

### Lutein Content Before and After Pelleting

The basal diets (control) before and after pelleting (powder and pellet) and the diets of the two lutein experimental groups were collected to determine the content of natural lutein. Before pelleting, the lutein contents of the control, lutein 1 and lutein 2 groups were 6.8, 20.4, and 30.5 mg/kg, respectively; after pelleting, the lutein contents of the control, lutein 1 and lutein 2 groups were 6.4, 19.4, and 29.1 mg/kg, respectively (Supplementary Table S3).

### Growth Performance and Skin Color of Yellow-Feather Chickens Between Days 53 and 74

Supplementary Table S4 shows no significant difference in the body weight between groups of 53-day-old animals (Supplementary Figure S1). Among the 60-day-old animals, the 1.5 g/kg lutein supplementation group had the heaviest body weight, but no significant differences were found between the groups in other traits (Supplementary Table S5). Among 67-day-old animals, the control group had the smallest hip, breast, leg, abdominal fat and shin b^*^ value (yellowness) and the 1.5 g/kg lutein supplementation group had the largest breast, leg and abdominal fat yellowness (Supplementary Table S6). Among the 74-day-old animals, the 1.5 g/kg lutein supplementation group had the largest hip, back, breast and leg yellowness, and the control group had the smallest hip, back, breast, leg, abdominal fat and shin yellowness (Supplementary Table S7).

**Figure 1:**
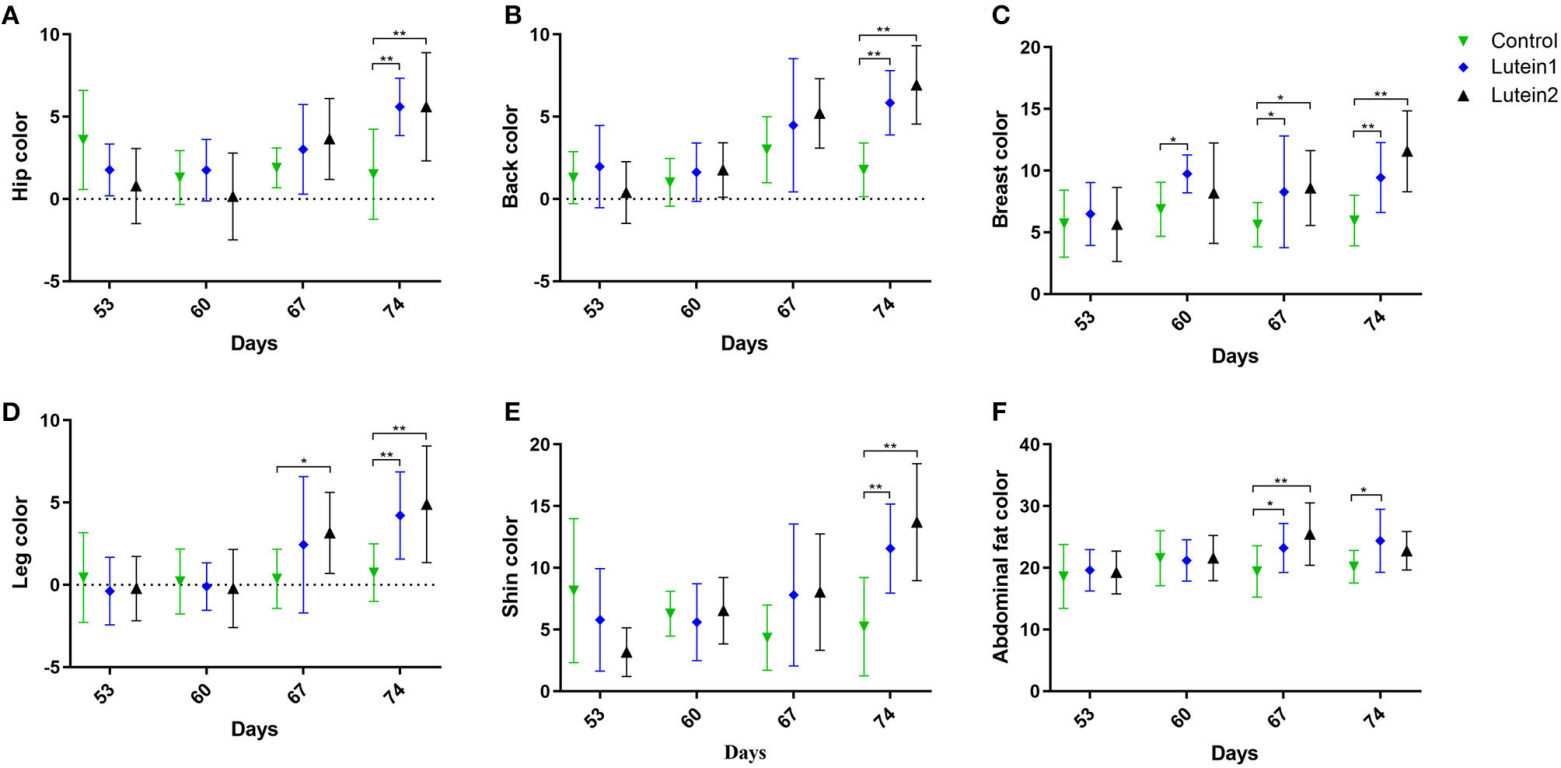
Yellowness measurement of yellow-feather chickens at 53, 60, 67 and 74 days of age. **(A)** Yellowness of the hip. **(B)** Yellowness of the back. **(C)** Yellowness of the leg. **(D)** Yellowness of the breast. **(E)** Yellowness of the abdominal fat. **(F)** Yellowness of the shin. *P < 0.05; **P < 0.01; *n* = 10.

Compared with the control group, at 67 days of age, the lutein 1 group had relatively higher yellowness of breast, leg and abdominal fat, and the lutein 2 group had relatively higher yellowness of breast and abdominal fat. At 74 days of age, both the lutein 1 and lutein 2 groups had relatively higher yellowness of the hip, back, breast, legs and shin than the control group, and the lutein 1 group had relatively higher abdominal fat yellowness. No significant difference was found in the yellowness of abdominal fat between the lutein 2 and control groups (Figure 1). The yellowness of the hip, back, breast, legs and shin of the lutein 1 and lutein 2 groups increased with increasing age compared with that of the control group, except for the yellowness of chicken abdominal fat (Supplementary Figure S2). By contrast, the yellowness of the hip, back, breast, legs and shins of the control group decreased with increasing age.

### ALT and AST Levels in Plasma

Compared with the control group, the plasma ALT levels in the lutein 1 and lutein 2 groups were significantly decreased, but no significant difference was observed in the AST levels between the groups (Supplementary Figure S3).

### Detection of SOD, GSH-PX and MDA Levels in Yellow-Feather Chickens

**Figure 2:**
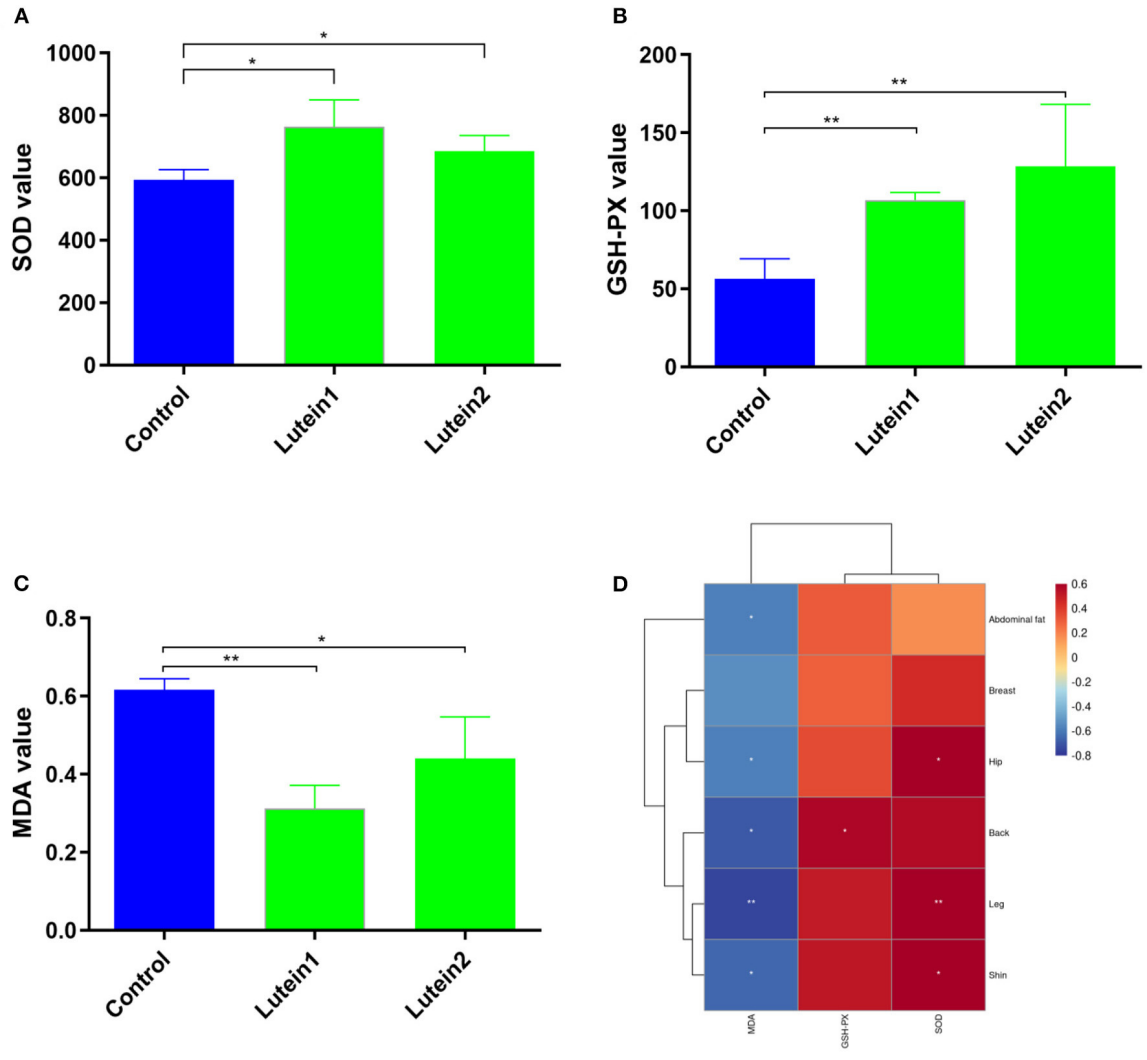
Determination of the liver antioxidant index. **(A)** SOD value of the liver. **(B)** GSH-PX value of the liver. **(C)** MDA value of the liver. **(D)** Correlation between skin yellowness and antioxidant indices. The color from blue to red indicates a negative correlation to a positive correlation, *P < 0.05; **P < 0.01.

The SOD levels in the lutein 1 group were significantly higher than those in the control group and no significant difference was found between the lutein 2 and control groups (Figure 2A). The GSH-PX levels in the control group were extremely significantly lower than those in the lutein 1 and lutein 2 groups (Figure 2B). The MDA value of the control group was extremely significantly higher than that of the lutein 1 group and significantly higher than that of the lutein 2 group (Figure 2C). The MDA level was significantly negatively correlated with the yellowness of the chicken abdominal fat, hip, back, leg and shin, the GSH-PX level was significantly positively correlated with the yellowness of the back, and the SOD level was significantly positively correlated with the yellowness of the hip, leg and shin (Figure 2D). These results consistently suggest that chickens fed lutein with higher phenotypic yellowness values may have higher antioxidant potential.

### Histopathological Evaluation

Hematoxylin and eosin staining of liver tissue showed that the structure and morphology of hepatocytes in the control and lutein 2 groups were normal, the boundary of the hepatic platelets was clear, and no obvious pathological change was observed (Supplementary Figure S4).

### Sequencing Results and Quality Control

**Figure 3:**
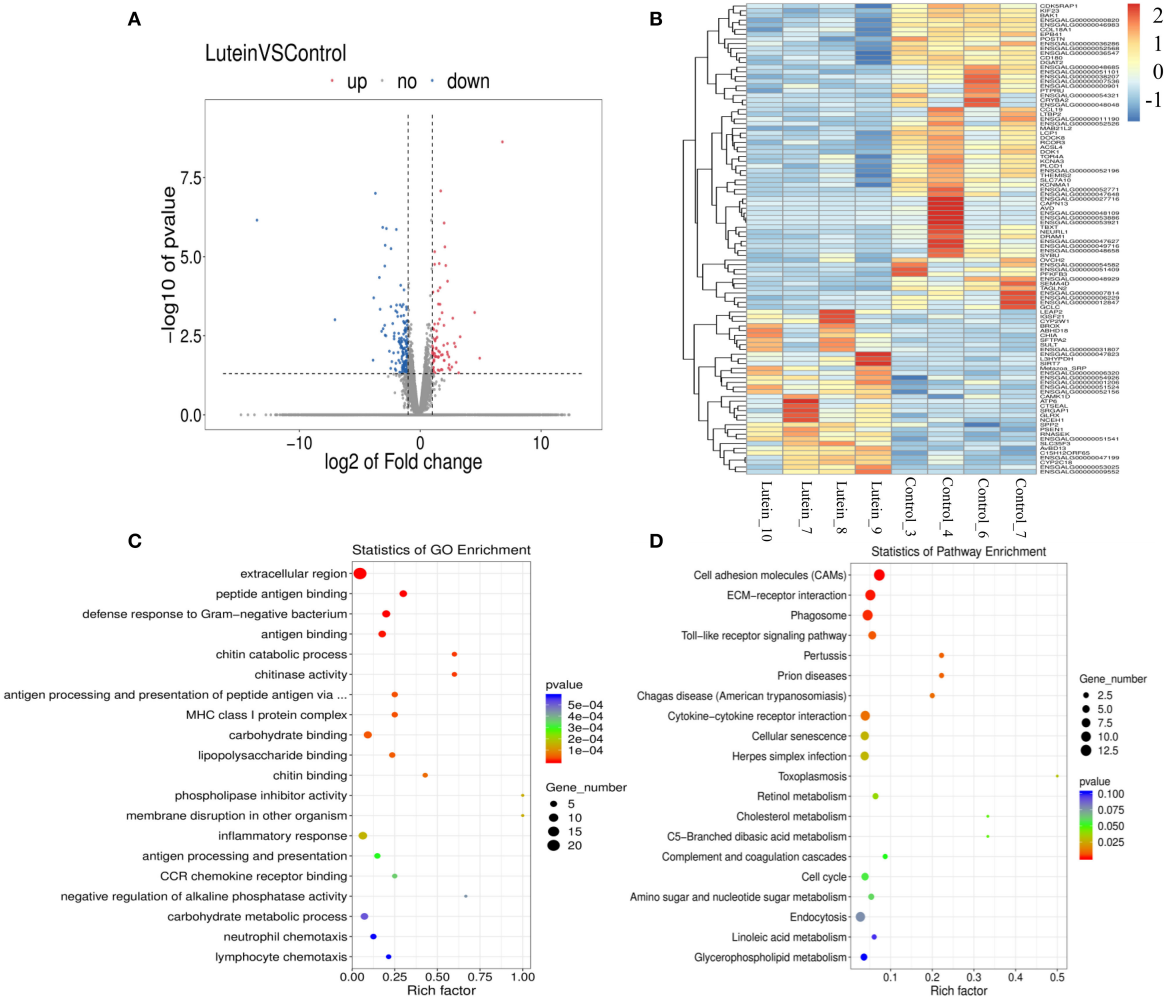
Expression profiles of differentially expressed genes (DEGs) and GO and pathway analysis. **(A)** Volcano plot of the significant differences in the DEGs. The horizontal and vertical axes represent the fold-change (FC) and *P-value*, respectively (FC > 2 or FC <0.5 and *P-value* < 0.05). **(B)** Heatmap of the expression profiles of all DEGs. The rows and columns represent the DEGs and samples, respectively. The color from blue to red indicates the log_10_ (FPKM + 1) values were from small to large, the red color indicates a high expression level, and the blue color indicates a low expression level. **(C)** Top 20 GO enrichment terms of all DEGs. **(D)** Top 20 KEGG pathways of all DEGs.

To identify the molecular regulatory mechanism of lutein in chickens, eight cDNA libraries were sequenced from the liver tissue of the lutein and control groups (four biological replicates). In total, 414,243,150 raw reads and 399,757,286 clean reads were generated, the percentages of clean reads among the raw reads were 96.26%−96.93%, and the average GC content of the clean reads was ~50% (Supplementary Table S8). Among the clean reads, 93.32%−94.54% mapped perfectly to the chicken reference genome, 89.41%−91.73% mapped to exons, 4.90%−6.86% mapped to introns, and 3.18%−3.73% mapped to intergenic regions (Supplementary Table S9). By comparing the sequencing data of the lutein and control groups, 155,547 transcripts were identified. Subsequently, the DEGs were screened based on the difference multiples FC ≥2 and *P* < 0.05. A total of 243 DEGs were identified between the groups, 80 upregulated and 163 downregulated genes (Supplementary Table S10; Figure 3A). Cluster analysis of DEGs revealed that the gene expression patterns of DEGs were clustered within groups, while the difference was significant between groups (Figure 3B).

### GO Enrichment Analysis

Gene Ontology functional annotation was performed on all the DEGs, revealing that these genes were significantly enriched in the three major categories of biological processes, cellular components, and molecular functions (Supplementary Figure S5). Overall, the top four GO terms were extracellular region, peptide antigen binding, defense response to Gram-negative bacterium and antigen binding; 22 genes were significantly enriched in the extracellular region of GO term. Three terms were related to chitin, including chitin catabolic process, chitinase activity, and chitin binding (Figure 3C; Supplementary Table S11). Except for a few genes enriched in oxidation-reduction process and cell redox homeostasis terms, the current functional annotation of the reference database does not contain redox-related differential genes.

### KEGG Pathway Enrichment Analysis

Kyoto Encyclopedia of Genes and Genomes pathway enrichment analysis was performed on the 243 DEGs. The top four significantly enriched pathways were cell adhesion molecules (CAMs), ECM-receptor interaction, and phagosome and Toll-like receptor signaling pathways. The top 20 pathways included cytokine–cytokine receptor interaction, cellular senescence, retinol metabolism, cholesterol metabolism and cell cycle signaling pathways (Figure 3D; Supplementary Table S12).

### Validating DEGs by QRT–PCR

**Figure 4:**
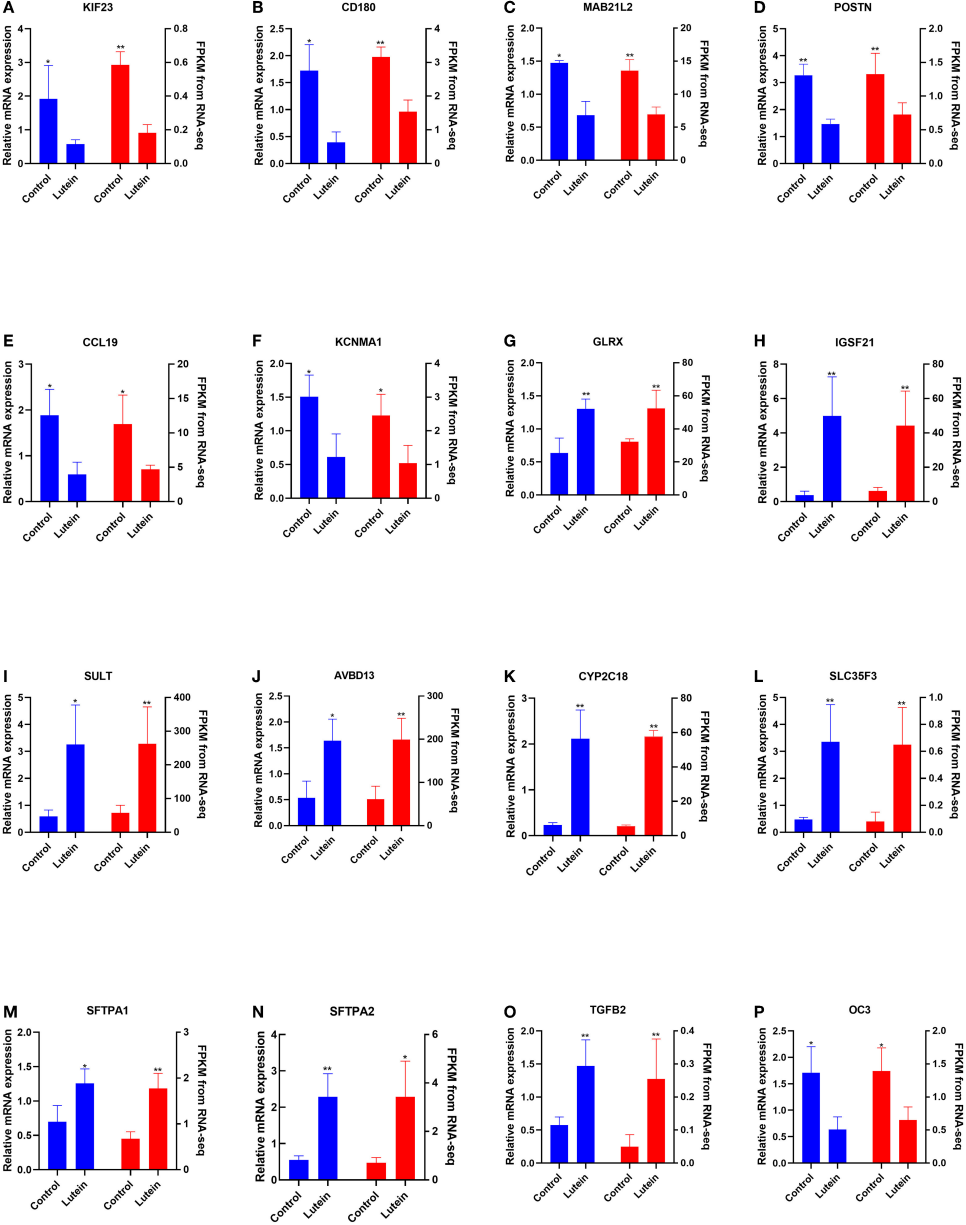
Comparison of the gene expression of RNA-seq with qPCR. The left and right Y axes represent the expression levels verified by qPCR and gene expression using FPKM units by RNA-seq, respectively. The blue column indicates qPCR; the red column indicates the FPKM value. The data represent the relative mRNA expression and FPKM of **(A)** *KIF23*, **(B)** *CD180*, **(C)** *MAB21L2*, **(D)** *POSTN*, **(E)** *CCL19*, **(F)** *KCNMA*, **(G)** *GLRX*, **(H)** *IGSF21*, **(I)** *SULT*, **(J)** *AvBD13*, **(K)** *CYP2C18*, **(L)** *SLC35F3*, **(M)** *SFTPA1*, **(N)** *SFTPA2*, **(O)** *TGFB2*, and **(P)** *OC3*.

The expression of 12 randomly selected DEGs was validated by qRT–PCR (qPCR). Compared with the control group, the kinesin family member 23 (*KIF23*), CD180 molecule (*CD180*), mab-21 like 2 (*MAB21L2*), periostin (*POSTN*), C-C motif chemokine ligand 19 (*CCL19*), potassium calcium-activated channel subfamily M alpha 1 (*KCNMA1*) and osteocalcin-like protein OC3 (*OC3*) genes were significantly downregulated in the liver of the lutein group, and the glutaredoxin (*GLRX*), immunoglobin superfamily member 21 (*IGSF21*), sulfotransferase (*SULT*), avian beta-defensin 13 (*AVBD13*), cytochrome P450 family 2 subfamily C member 18 (*CYP2C18*), solute carrier family 35 member F3 (*SLC35F3*), surfactant protein A1 (*SFTPA1*), surfactant protein A2 (*SFTPA2*) and transforming growth factor beta 2 (*TGFB2*) genes were significantly upregulated in the liver of the lutein group. The expression of these genes was consistent with the RNA-seq results (Figures 4A–P).

### Metabolic Profiling of Chicken Serum

The extracted substances were analyzed by LC–MS of the untargeted metabolome, and the positive ion and negative ion modes were tested. Quality control of the extracted substances was performed using XCMS software, and the QC sample and total ion chromatogram (TIC) were compared with overlapping spectra. The response intensity and retention time of each chromatographic peak overlapped, indicating that the variation caused by instrument error during the whole experiment was small and the data quality was reliable (Supplementary Figures S6A,B).

**Figure 5:**
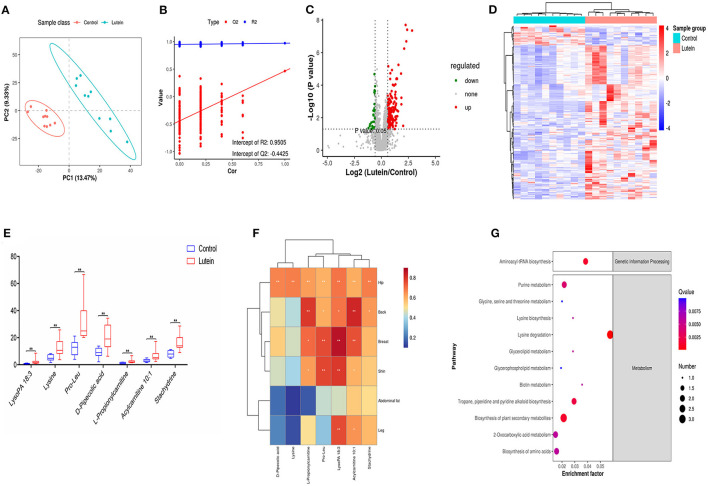
Metabolome changes induced by lutein. **(A)** PLS-DA score plots in chicken plasma induced by lutein. Each point represents a sample, and the degree of dispersion of the two colors represents the distribution trend of the two groups on the PC1 and PC2 axes. **(B)** Permutation test of the PLS-DA model. The number of random permutation tests is 200. **(C)** Volcano map of different metabolites. The horizontal axis represents the fold change of metabolic ions in the comparison group, and the vertical axis shows –log_10_ (*q*-value). **(D)** Heatmap of the relative abundance of differential metabolites (VIP ≥ 1, *q*-value ≤ 0.05). Red indicates high relative abundance, and blue indicates low relative abundance. **(E)** Box plot of potential metabolites in the two groups. **P < 0.01. **(F)** The correlation between skin yellowness and SDMs. The color from blue to red indicates a negative correlation to a positive correlation, *P < 0.05, **P < 0.01. **(G)** KEGG pathway of differential metabolites.

To confirm the high quality of the data, quality control was performed by inserting QC samples. The PCA results showed that the 4 QC samples were clustered in the middle of all samples, indicating that the data quality of this experiment was high, and all the samples were within the 95% confidence interval (Supplementary Figure S6C). PLS-DA is a supervised difference discriminant analysis method used to screen potential SDMs. PLS-DA analysis showed that the plasma samples were significantly separated into the control and lutein groups (Figure 5A). At the same time, the parameters R2 and Q2 of the PLS-DA model were subjected to permutation tests (the number of tests was 200). The value of R2 was close to 1, indicating that the established model met the real situation of the sample data; Q2 <0 indicated that the PLS-DA model was reliable without overfitting (Figure 5B).

### Screening and Identification of SDMs

We analyzed the annotated metabolites in plasma. Compared with the control group, the lutein group annotated 59 SDMs in the negative mode (49 upregulated metabolites and 10 downregulated metabolites) and 178 SDMs in the positive mode (161 upregulated and 17 downregulated metabolites; Supplementary Table S13; Figure 5C). According to the substance information, the SDMs were classified into 24 types. In the positive and negative ion modes, lipids and lipid-like molecules had the largest number of metabolites, 29,398 and 6,846, respectively (Supplementary Figure S6D). Hierarchical cluster analysis was used to identify the change characteristics of the SDMs, revealing that the different metabolites of the two groups of samples were significantly different (Figure 5D). Next, using the standard ratio >2 or ratio <0.05, *P* < 0.01, seven SDMs were screened out. These potential metabolites were considered biomarkers, and the relative concentration changes of these metabolites are shown in Figure 5E. The concentration of the seven candidate metabolites had a significant positive correlation with the yellowness of the chicken hip, and the concentration of the five metabolites had a significant positive correlation with the yellowness of the chicken back. In addition to the yellowness of the abdominal fat, LysoPA 18:3 and acylcarnitine 10:1 had a significant positive correlation with the yellowness of the hip, back, breast, leg and shin (Figure 5F).

To further explain the biological functions of metabolites, pathway enrichment was performed based on KEGG annotation, and enrichment significance analysis was performed. However, the annotation based on KEGG has a certain degree of limitation, many metabolites cannot be enriched in the KEGG pathway. As shown in Figure 5G, the significantly changed candidate biomarkers were distributed in different metabolic pathways, including aminoacyl-tRNA biosynthesis; glycine, serine and threonine metabolism; lysine biosynthesis and lysine degradation; glycerolipid metabolism; and glycerophospholipid metabolism. Among the first 20 enrichment pathways screened, metabolic pathways, biosynthesis of secondary metabolites and lysine degradation pathway showed the most enrichment of metabolites. Six pathways were related to amino acid metabolism, namely, lysine degradation, arginine and proline metabolism, cysteine and methionine metabolism, glycine, serine and threonine metabolism, lysine biosynthesis, and phenylalanine metabolism pathways. In particular, l-lysine metabolites were enriched in 11 pathways, l-methionine metabolites were enriched in nine pathways, 1-acyl-sn-glycerol 3-phosphate was enriched in four pathways, and creatine was enriched in three pathways. The pathways shared by the four metabolites were metabolic pathways (Figure 5G; Supplementary Table S14).

### Network Analyses for the SDMs and DEGs

**Figure 6:**
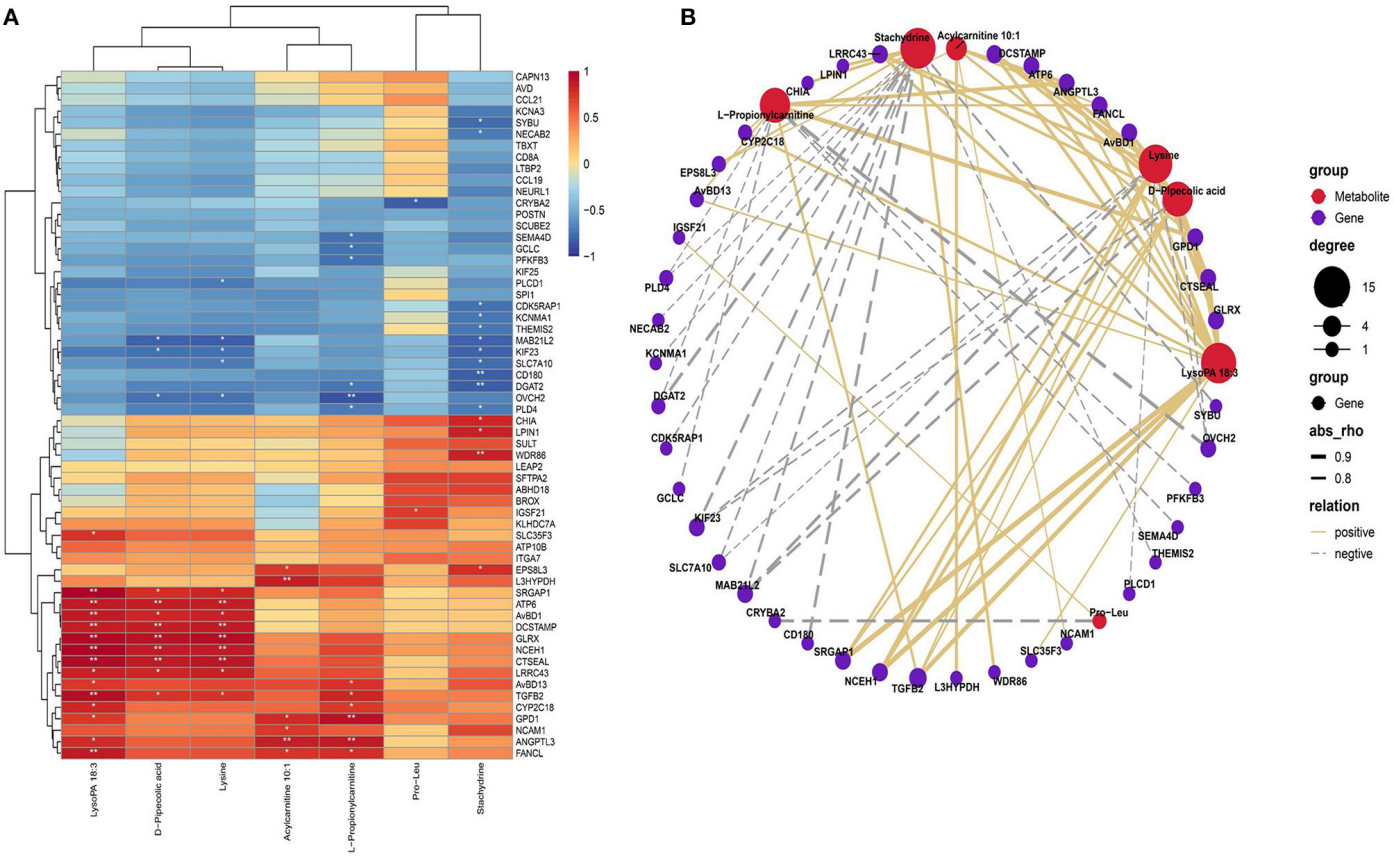
Comprehensive analysis of the SDMs and DEGs. **(A)** Correlation heatmap of differentially expressed genes and differential metabolites. Color from blue to red indicates a negative correlation to a positive correlation, *P < 0.05, **P < 0.01. **(B)** Correlation network diagram of differentially expressed genes and differential metabolites. Only the 20 upregulated and downregulated DEGs are displayed, and the differential metabolites of the top seven are displayed.

Based on the Pearson correlation analysis of SDMs and DEGs, clustering correlation heatmap with signs was performed using the stats package. The heatmap results showed that LysoPA 18:3 has a highly significant positive correlation with genes such as *GLRX* and neutral cholesterol ester hydrolase 1 (*NCEH1*). Acylcarnitine 10:1 has a highly significant positive correlation with trans-l-3-hydroxyproline dehydratase (*L3HYPDH*) and angiopoietin like 3 (*ANGPTL3*), and l-propionylcarnitine has a highly significant positive correlation with glycerol-3-phosphate dehydrogenase 1 (*GPD1*) and *L3HYPDH*. LysoPA 18:3, d-pipecolic acid and l-lysine were highly positively correlated with genes such as ATP synthase F0 subunit 6 (*ATP6*), *GLRX*, and *NCEH1*. Stachytine is highly negatively correlated with the CD180 molecule (*CD180*) and glycerol *O*-acyltransferase 2 (*DGAT2*; Figure 6A).

We used the igraph software package (version 1.2.6) to further analyze the relationship between altered metabolites in plasma and genes in the liver that formed an association network (Figure 6B). The correlation network showed that LysoPA 18:3, lysine and d-pipecolic acid were positively correlated with genes such as *NCEH1*, transforming growth factor beta 2 (*TGFB2*), *GLRX, ATP6*, and avian beta-defensin 1 (*AVBD1*); lysine, d-pipecolic acid and stachydrine were negatively correlated with *KIF23* and *MAB21L2*. Additionally, stachydrine was negatively correlated with *CD180, DGAT2* and other genes and positively correlated with chitinase acidic (*CHIA*) and other genes. l-Propionylcarnitine was positively correlated with *GPD1* and *TGFB2* and other genes and negatively correlated with *DGAT2* and other genes. Pro-Leu was positively correlated with *IGSF21* and negatively correlated with crystallin beta A2 (*CRYBA2*).

## Discussion

Adding marigold extract (lutein) to the diet can significantly increase the yellowness b^*^ value of broiler tarsals, beaks, skin and muscles, and the yellowness value is positively correlated with the amount of marigold extract added (28). Figure 1 shows that lutein supplementation significantly improved the overall skin color and yellowness of abdominal fat of the yellow-feather chickens at 67 days and 74 days of age, respectively. Supplementary Figure S2 shows that the overall skin color growth rate of the lutein supplementation group gradually increased over time, while the overall skin color growth rate of the control group gradually decreased or did not change. These results consistently showed that lutein could improve the overall yellowness of chicken skin to a certain extent and corroborate the results of previous studies (29). Additionally, the effect of lutein supplementation was most pronounced on the 21st day of feeding.

Previous studies have shown that dietary supplements of lutein and/or zeaxanthin have resulted in rapid increases in plasma or serum concentrations of these carotenoids in BALB/c mice, quail, and Rhesus monkeys (24). Similar to other carotenoids, lutein and zeaxanthin are incorporated into chylomicrons along with dietary lipids after intestinal absorption. Chylomicrons are rapidly remodeled by lipoprotein lipase in peripheral tissues and enter the circulation as chylomicron remnants. The resulting carotenoid-containing chylomicron residues are then transferred to the liver, where they can be stored or re-secreted into the circulation along with lipoproteins (30). Therefore, we speculate that circulating lutein may also play a potential role. Lutein in feed is usually deposited in the skin and fat of chickens (31). In recent studies, some researchers have proven that adding lutein to feed can improve the antioxidant activity of chickens. Compared with the control group, supplementation with 0.15 and 0.60% marigold extract can significantly improve the serum SOD, CAT and GSH-Px activities of 21-day-old broilers; in another study, 200 and 300 mg/kg of marigold supplementation increased SOD and GSH-Px activities significantly, while the MDA content decreased significantly (29, 32). Our results also showed that lutein 2 supplementation significantly increased the SOD and GSH-Px activities in the liver and significantly reduced the MDA content in chickens (Figure 2). At the cellular level, oxidative stress can reduce cell viability and increase total apoptosis and ROS production. However, lutein can prevent cell damage induced by oxidative stress (8). These results indicate that lutein may be a potential antioxidant in animals. Interestingly, the correlation between the values of MDA, GSH-PX and SOD and yellowness of chicken skin indicates that the higher is the value of skin yellowness in chicken, the higher is the antioxidant capacity; however, this result requires further experimental verification. ALT and AST are commonly used biomarkers for liver injury. In rats, alcohol can significantly increase serum ALT and AST, and lutein treatment can significantly reduce these levels (33). In the present study, lutein decreased ALT enzyme release in plasma, suggesting that lutein may also improve liver function.

Gene Ontology analysis showed that DEGs were significantly enriched in certain cellular components, molecular functions and biological processes. DEGs are important in understanding how lutein regulates liver metabolism. In our study, 243 DEGs were obtained in the lutein and control groups using RNA-Seq. Some of these gene products may participate in lipid metabolism and the conversion of carotenoids. For example, the expression of the *CCL19* gene was significantly reduced in the lutein group. Compared with wild-type mice, *CCL19* knock-in mice showed increased inflammation in adipose tissue and increased subcutaneous white and brown adipose tissue (34). The expression of the *CCL19* gene is increased in obese human adipose tissue and is positively correlated with markers of insulin resistance and metabolism, indicating that *CCL19* may be an essential marker for predicting insulin resistance and metabolism (35). Additionally, a study has also shown that the pathway formed by the *CCL19* gene and its receptor CCR7 plays a key role in inducing obesity and insulin resistance (36). Many previous studies investigated oxidative stress involving the cytokine–cytokine receptor interaction signaling pathway, and *CCL19* was enriched in this pathway, suggesting that *CCL19* and oxidative stress also have a potential relationship (37, 38).

*GLRX* is a critical oxidoreductase and pleiotropic cytokine that plays a role in cell growth, apoptosis, and inflammation and protects cells from oxidative stress (39, 40). *GLRX* controls lipid homeostasis by regulating protein GSH adducts in the liver. Compared with wild-type mice, *GLRX* knockout mice became obese at 8 months, their body weight and relative fat mass ratio increased significantly, the expression of fatty acid metabolism-related genes in the liver of *GLRX* knockout mice was significantly increased, and the plasma triglyceride and cholesterol levels were significantly elevated (41). *GLRX* knockdown significantly reduced the GSH/GSSG ratio in human cell lines, resulting in excessive ROS accumulation and activation of p53 and related signaling pathways, while *GLRX* overexpression reduced cellular ROS levels (42). *NCEH1* encodes a key enzyme that inhibits the formation of lipid droplets by removing cholesterol in macrophage foam cells (43). Conversely, ablation of *NCEH1* promotes foam cell formation and atherosclerosis in mice (44). *DGAT2* plays a key role in the synthesis of triglycerides in animals and is highly expressed in the liver and white adipose tissue of mice (45, 46). In chicken embryonic fibroblasts (CEFs), lipid accumulation and the expression of genes such as *DGAT2* can be increased by simultaneously supplementing fatty acids and insulin to induce adipogenic differentiation for 48 h (47). In this study, the expression of *DGAT2* in chicken liver was significantly downregulated after lutein feeding, indicating that this gene may be related to liver lipid metabolism.

Defensins play a vital role in the innate immune response, and they are expressed in birds (48). Compared with the control group, the expression of the β-defensin-1 (*AvBD1*) and *AvBD13* genes in the liver of the lutein group was significantly increased, suggesting that lutein may improve the innate immunity of chickens. Previous studies have shown similar results. Dietary lutein has anti-inflammatory effects and can regulate the cat's humoral immune response (49, 50).

Lysine is an essential amino acid critical for normal growth in animals (51). The addition of lysine can significantly reduce the level of ROS induced by high glucose in C2C12 myotubes and 3T3-L1 adipocytes and can regulate the unfolded protein response and autophagy (52). Previous studies have shown that d-pipecolic acid in plasma originates from the catabolism of dietary lysine in the intestine. The level of d-pipecolic acid in plasma increased significantly in humans 2 h after soybean juice was ingested, and the level of lysine in plasma showed an increase similar to that of d-pipecolic acid (53). Importantly, our plasma metabolome also showed similar results, with the same trends for lysine and d-pipecolic acid. Stachydrine can significantly inhibit the expression of *IL-6, IL-8, IL-1*β, tumor necrosis factor-α and other related inflammatory factors in the liver. Additionally, oxidative stress is inhibited by reducing the level of malondialdehyde in the serum and increasing the level of GSH and enzymatic activities of CAT, glutathione reductase, SOD and GSH-PX (54). In our study, strachydrine metabolites were significantly negatively correlated with *KCNMA1* and *DGAT2*. Previous study findings have shown that *DGAT2* is related to liver lipid metabolism; *KCNMA1* was identified as a candidate gene for obesity by genome-wide association analysis (55). These results suggest that stachydrine may be related to liver lipid metabolism.

The water-soluble glycerophospholipid lysophosphatidic acid (1- or 2-acyl-sn-glycerol 3-phosphate, LPA, LysoPA) is a candidate mediator of adipocyte differentiation. LPA exists in blood and body fluids and can play extracellular roles through signal transmission of the G protein-coupled receptor family (56, 57). Additionally, LPA can promote preadipocyte proliferation and inhibit differentiation by TG accumulation and PPARγ expression (58, 59). Many studies have also proven that lysophosphatidic acid (LysoPA) is an effective ROS regulator (60). In HEK293 cells, activation of the lysophosphatidic acid receptor LPA3 increases the expression level of antioxidant enzymes, inhibiting ROS accumulation and improving cell senescence (61). In the present study, the level of LysoPA was significantly positively correlated with the expression of the *NCEH1* gene, and LysoPA was enriched in the glycerolipid metabolism and glycerophospholipid metabolism pathways. Interestingly, the common pathway for plasma SDMs and liver DEGs is glycerophospholipid metabolism. These results show that LysoPA may be strongly correlated with liver lipid metabolism and antioxidation.

## Conclusion

In summary, our study showed the overall changes in liver transcripts and plasma metabolites of chickens fed lutein. Our findings show that lutein can significantly increase the antioxidant level and skin yellowness in yellow-feather chickens and may help improve the health of chickens. DEGs in the liver and SDMs in plasma have potential effects on the lipid metabolism and antioxidant processes of chickens. However, the detailed regulatory mechanism must be further studied and verified.

## Data Availability Statement

All the data in the current study can be obtained from the corresponding author upon reasonable request. The raw reads were deposited into the Sequence Read Archive (SRA) database (PRJNA785785). Metabolome data reported in this paper have been deposited in the OMIX database of NGDC (https://ngdc.cncb.ac.cn/omix: accession no. OMIX001093).

## Ethics Statement

All the experimental procedures in this study were performed according to the Institutional Animal Care and Use Committee guidelines and were approved by South China Agricultural University (approval number: SCAU-2018f052).

## Author Contributions

TR performed the experiments, analyzed the data, and wrote the manuscript. WL collected the samples and performed the experiments. SH and XY performed the additional experiments. MX and ZZ collected the samples. WL and QN revised the manuscript. XZ designed this experiment and reviewed the manuscript. All the authors agreed to approve the final manuscript.

## Funding

This study was supported by the China Agriculture Research System (Grant No. CARS-41-G03), and the Science and Technology Program of Guangzhou, China (Grant No. 201804020088).

## Conflict of Interest

The authors declare that the research was conducted in the absence of any commercial or financial relationships that could be construed as a potential conflict of interest.

## Publisher's Note

All claims expressed in this article are solely those of the authors and do not necessarily represent those of their affiliated organizations, or those of the publisher, the editors and the reviewers. Any product that may be evaluated in this article, or claim that may be made by its manufacturer, is not guaranteed or endorsed by the publisher.
