# Supplementary material for: Integrative Analysis of Metabolomic and Transcriptomic Data Reveals the Antioxidant Potential of Dietary Lutein in Chickens
Source: Front Vet Sci. 2022 Jun 23;9:906853. doi: 10.3389/fvets.2022.906853 (PMC9260106; doi:10.3389/fvets.2022.906853)
Supplement: Supplementary file 1 [file Data_Sheet_1.ZIP › Supplementary information/Supplementary Materials.docx]

**Integrative Analysis of Metabolomic and Transcriptomic Data Reveals the Antioxidant Potential of Dietary Lutein in Chickens**

Tuanhui Ren^1,2^, Wujian Lin^1,2^, Shizi He^1,2^, Xiuxian Yang^1,2^, Mingjian Xian^1,2^, Zihao Zhang^1,2^, Wen Luo^1,2^, Qinghua Nie^1,2^, Xiquan Zhang^1,2*^


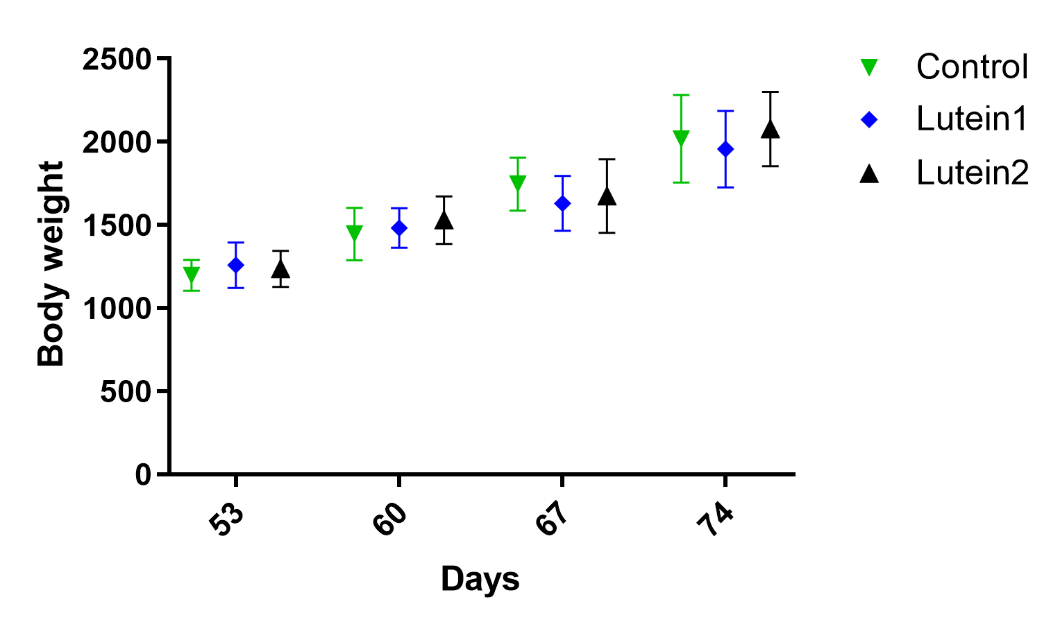


**Figure S1. Body weight of yellow-feather chickens at 53, 60, 67 and 74 days of age.**


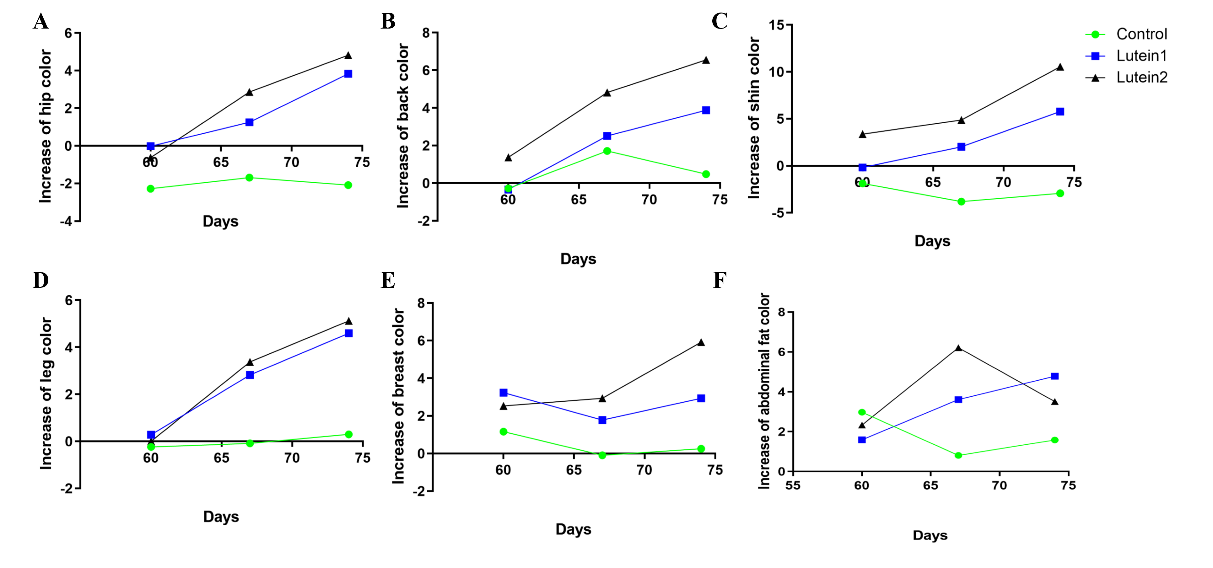


**Figure S2. Increasing trend of yellowness in yellow-feather chickens at 53, 60, 67 and 74 days of age.** (A) Yellowness of the hip. (B) Yellowness of the back. (C) Yellowness of the shin. (D) Yellowness of the leg. (E) Yellowness of the breast. (F) Yellowness of the abdominal fat. N=10 chickens per group.


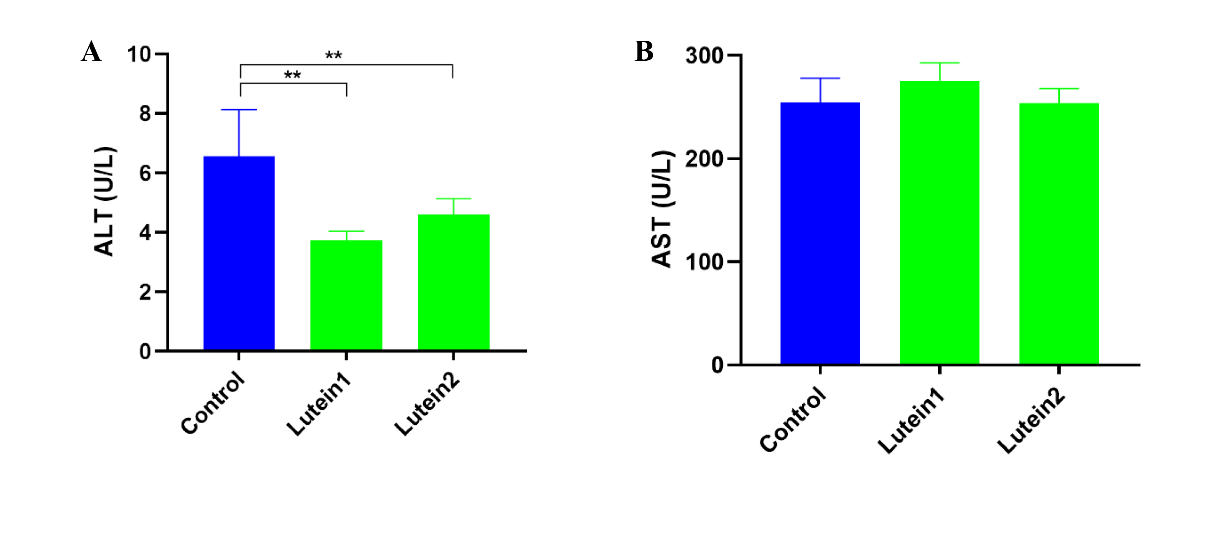


**Figure S3. Determination of plasma ALT and AST.** (A) ALT levels in plasma. (B) AST levels in plasma. ** *P* < 0.01.


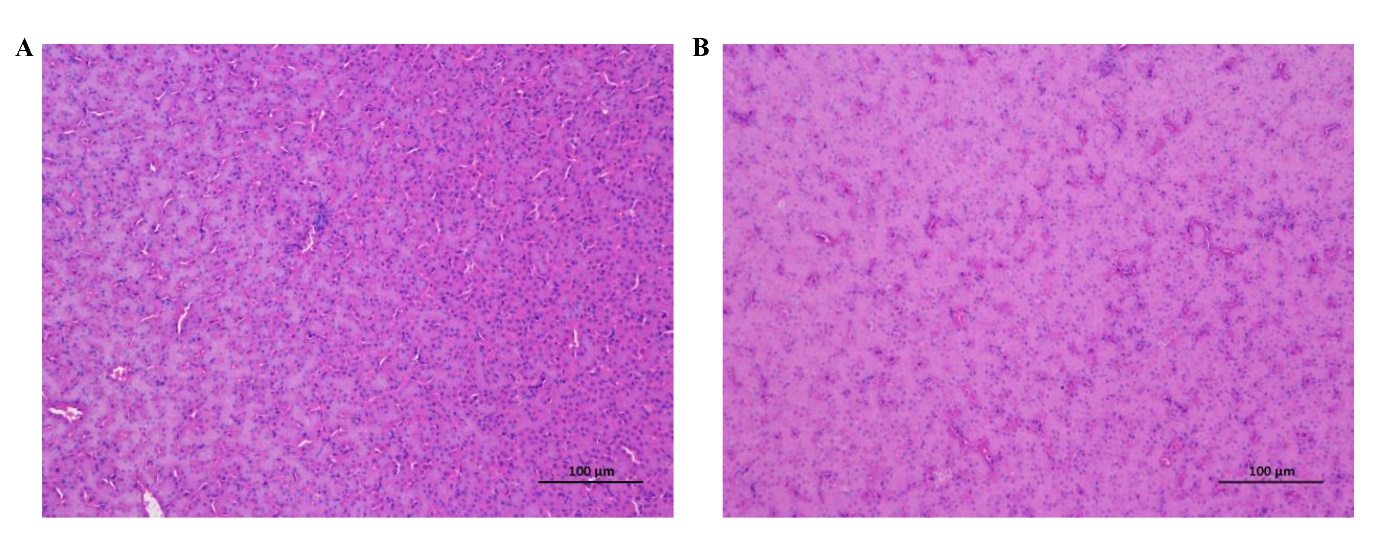


**Figure S4. Liver histopathological sections by HE staining (×200).** (A) Control group. (B) Lutein 2 group.


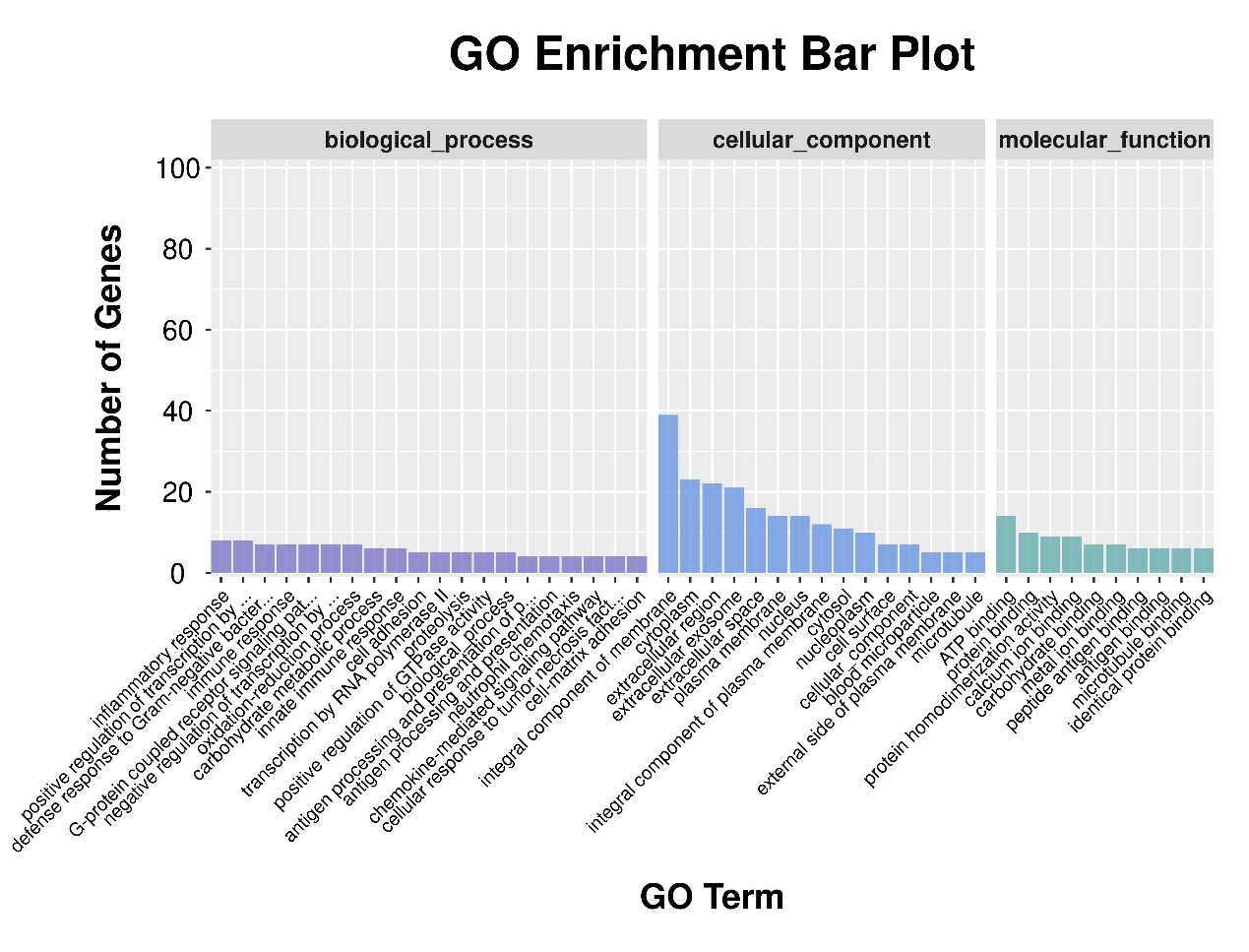


**Figure S5. The GO function annotation all DEGs.**


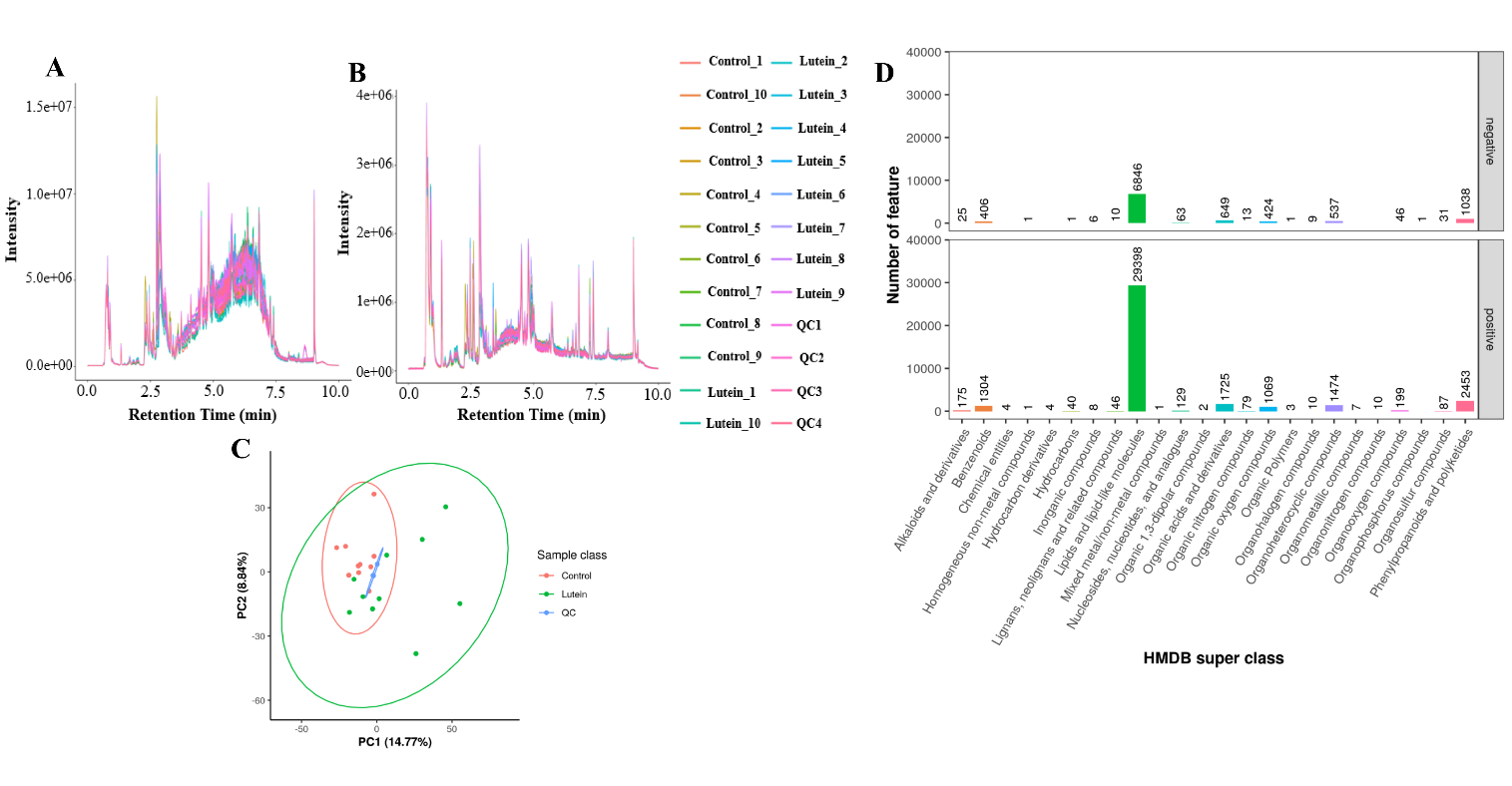


**Figure S6.** Metabolome analysis in plasma. (A, B) Total ion chromatogram. The horizontal axis is the time point, the vertical axis is the total intensity of all ions in the mass spectrum at each time point, and each color represents a sample. (C) Principal component analysis (PCA) of metabolites. Each point represents a sample in the figure, and the similarities and differences between all samples are reflected in the separation and aggregation trends of the samples in the figure. (D) HMDB Super class. The horizontal axis represents the super class entry, and the vertical axis represents the number of metabolites in the corresponding entry.

**Table S1.** Composition and nutrient level of basal diets (air-dry basis, %).

|  | Diets | | |
| --- | --- | --- | --- |
| Ingredients/% | 1-20 days old | 21-40 days old | 41-84 days old |
| Corn | 54.80% | 58.60% | 62.30% |
| Wheat middlings | 5.00% | 3% | 2% |
| Soybean oil | 1.50% | 4% | 5% |
| Soybean meal | 31% | 28% | 21.50% |
| Corn protein meal | 2% | 2% | 2% |
| Sunflower meal |  |  | 3% |
| Fish meal | 1% |  |  |
| limestone | 1.50% | 1.40% | 1.30% |
| Calcium hydrogen phosphate | 1.20% | 1% | 0.90% |
| Premix | 2% | 2% | 2% |
| Total | 100% | 100% | 100% |
| Nutrient levels |  |  |  |
| MC/kg | 2.95 | 3.15 | 3.2 |
| CP | 21% | 19% | 17.50% |
| Ca | 1.05% | 0.92% | 0.85% |
| P | 0.48% | 0.42% | 0.39% |
| Lys | 1.18% | 1% | 0.95% |
| Met | 0.54% | 0.45% | 0.42% |
| Thr | 0.79% | 0.72% | 0.65% |
| Trp | 0.24% | 0.21% | 0.18% |
| Na | 0.16% | 0.16% | 0.15% |
| Cl | 0.20% | 0.20% | 0.20% |

**Table S2.** DEGs primers used for RT–qPCR.

| Gene name | Primer sequences (5'-3') | Sizes (bp) | Tm (℃) |
| --- | --- | --- | --- |
| KIF23 | F: GAAGGGAACAGGTTGCGAGA | 123 | 60 |
|  | R: ATCCCTGTAGGGCACCATCT |  |  |
| POSTN | F: CTGCAGGTGGTACGGACAAT | 226 | 60 |
|  | R: GGTGCTTCTTCCAAACGGAC |  |  |
| SULT | F: CAATGGATCGTGATGAACTGCT | 187 | 60 |
|  | R: AATGTGGCCACCAGATCACTT |  |  |
| GLRX | F: AGCAGAGTTCCTGCTCAACC | 103 | 60 |
|  | R: GGCAGGATCCCTTCACGAAA |  |  |
| CD180 | F: ACAGCCTTGGGAAGTTACGG | 199 | 60 |
|  | R: CAGGTGCATTCCAGAGGGTT |  |  |
| CCL19 | F: CAGGGGCTTCTTGACAGGAG | 182 | 60 |
|  | R: GCTCGAGGCTGATGATGGAA |  |  |
| AvBD13 | F: CAGCTGTGCAGGAACAACCA | 59 | 60 |
|  | R: CAGCTCTCCATGTGGAAGCA |  |  |
| SLC35F3 | F: CGGGTTGACTTTGAAGGTGT | 193 | 60 |
|  | R: CCTCACTCCCATGAACCTGT |  |  |
| MAB21L2 | F: CTGCTGTACGAGTGCGAGAA | 245 | 60 |
|  | R: AGTTTGTCGAGGCTTTTGGGA |  |  |
| KCNMA | F: TTAATGGTGGTGGAGATACCAGG | 118 | 60 |
|  | R: ATGACTCCTGCCCAGTCTTTC |  |  |
| CYP2C18 | F: GAGACTGCCCTTTGACCCAA | 152 | 60 |
|  | R: GGGAGTTCATCATCTCAAATGTGT |  |  |
| IGSF21 | F: GGTCTACCAGTCCACAGTGC | 144 | 60 |
|  | R: GGAGCCATCACGTTGAGGAA |  |  |
| SFTPA1 | F: TCTTTTGCTCCCCTGCTACG | 125 | 60 |
|  | R: TTTCGCTACCGGCAACTGAT |  |  |
| SFTPA2 | F: AAATGCAACCTGTACCGCCT | 73 | 60 |
|  | R: GGTCTCCATTCTCAGGTGCC |  |  |
| TGFB2 | F: GGCTGAGTTCAGGGTCTTCC | 211 | 60 |
|  | R: CCTGTCTCTGTGATGGAGCC |  |  |
| OC3 | F: AAGCTACCCTCCCTGTGACT | 138 | 60 |
|  | R: AAGATTCTTTCTGCCGGGGG |  |  |
| β-actin | F: GACTGACCGCGTTACTCCCA | 166 | 60 |
|  | R: CCAACCATCACACCCTGATGTC |  |  |

**Table S3.** Determination of the lutein content before and after pelleting (air-dried basis, %).

| Sample | Item | Result | Unit | Method |
| --- | --- | --- | --- | --- |
| Control (powder) | Lutein | 6.8 | mg/kg | GB/T 23187-2008 |
| Control (pellet) | Lutein | 6.4 | mg/kg | GB/T 23187-2008 |
| Lutein1 (powder) | Lutein | 20.8 | mg/kg | GB/T 23187-2008 |
| Lutein1 (pellet) | Lutein | 19.4 | mg/kg | GB/T 23187-2008 |
| Lutein2(powder) | Lutein | 30.5 | mg/kg | GB/T 23187-2008 |
| Lutein2 (pellet) | Lutein | 29.1 | mg/kg | GB/T 23187-2008 |

**Table S4.** Growth traits and yellowness value (b*) of 53-day-old yellow-feather chickens (Mean±SD).

**Table S5.** Growth traits and yellowness value (b*) of 60-day-old yellow-feather chickens (mean±SD).

**Table S6.** Growth traits and yellowness value (b*) of 67-day-old yellow-feather chickens (mean±SD).

**Table S7.** Growth traits and yellowness value (b*) of 74-day-old yellow-feather chickens (Mean±SD).

**Table S8.** Quality analyses of sequenced reads.

**Table S9.** Regional distribution of reference genome alignment.

**Table S10.** Gene expression data of RNA-seq from the lutein and control groups.

**Table S11.** GO enrichment terms of DEGs.

**Table S12.** KEGG enrichment pathway of DEGs.

**Table S13.** SDMs of plasma between the lutein and control groups.

**Table S14.** SDMs in the top 20 KEGG enrichment pathways.
